# The Transcription Factor SOX5 as a Potential Marker of Resistance to Chemotherapy in Gastric Cancer Cell Lines

**DOI:** 10.3390/ijms27209228

**Published:** 2026-10-17

**Authors:** Joaquín Reyes-González, Guillermo Valenzuela, Maher Musleh, Natalia Landeros, Héctor R. Contreras

**Affiliations:** 1 Laboratory of Molecular and Cellular Oncology, Department of Basic and Clinic Oncology, Faculty of Medicine, University of Chile, Santiago 8380453, Chile; 2 Center for Cancer Prevention and Control (CECAN), Santiago 8331150, Chile; 3 Department of Surgery, University of Chile Clinical Hospital, Universidad de Chile, Santiago 8380456, Chile; 4 Laboratorio de Genómica, Bioinformática y Biosensores Aplicados, Facultad de Medicina, Universidad Católica del Maule, Talca 3460000, Chile

**Keywords:** gastric cancer, SOX5, epithelial–mesenchymal transition, chemoresistance, fluorouracil, oxaliplatin

## Abstract

Chemoresistance is one of the major barriers to effective treatment in gastric cancer (GC), contributing to disease progression and poor patient survival. SRY-box transcription factor 5 (SOX5) has been associated with aggressive cancer phenotypes in several malignancies; however, its relationship with chemoresistance in GC has yet to be fully characterized. This study investigated whether SOX5 expression is associated with molecular features linked to aggressive GC phenotypes, including multi-drug resistance proteins, cancer stem cell (CSC) antigens, epithelial–mesenchymal transition (EMT) markers, and proteins involved in drug efflux and DNA repair. SOX5 expression was evaluated in AGS and MKN-45 GC cell lines using Western blotting, real-time quantitative PCR, and immunofluorescence. Markers associated with EMT (Snail1, ZEB1, and E-cadherin), CSC phenotypes (CD44 and CD133), DNA repair (XRCC1 and ERCC2), and drug efflux (ABCB1) were analyzed. Cellular sensitivity to 5-fluorouracil (5-FU) and oxaliplatin (Ox) was assessed using an MTT-based viability assay. In addition, publicly available GC datasets were analyzed to evaluate the association between SOX5 expression and overall survival. AGS cells exhibited significantly higher SOX5 expression at both the mRNA and protein levels compared with MKN-45 cells. High SOX5 expression was associated with increased expression of the transcription factors Snail1 and ZEB1, which are associated with EMT, leading to decreased E-cadherin expression. Furthermore, SOX5 expression was associated with increased expression of CD133, the DNA repair genes XRCC1 and ERCC2, and ABCB1 transcripts. Consistent with these molecular findings, AGS cells exhibited greater viability following exposure to 5-FU and Ox, either alone or in combination, suggesting reduced sensitivity to these agents. Furthermore, high SOX5 expression was associated with poorer overall survival across independent GC patient cohorts. Overall, these findings suggest SOX5 as a potential biomarker associated with an aggressive molecular phenotype characterized by EMT activation, enhanced DNA repair capacity, stemness-associated features, and reduced sensitivity to FOLFOX-based chemotherapy. Future mechanistic studies are warranted to establish whether SOX5 directly drives these processes.

## 1. Introduction

Gastric cancer (GC) is the fourth most common malignant neoplasm with the highest incidence and mortality [1]. Currently, the treatment of GC involves a combination of surgery, chemotherapy and radiotherapy based on the stage [2,3]. In advanced stages and with unresectable tumors, the first-line treatment is conventional chemotherapy [2]. Nevertheless, in advanced stages, survival declines significantly, with only 16.7% overall survival at 5 years [4]. This poor survival is largely because the response to chemotherapy is not sustained over time, mainly due to the development of chemoresistance [5].

The chemotherapeutic agents with the strongest evidence for efficacy in GC are 5-fluorouracil and oxaliplatin, particularly when both are combined with leucovorin in the FOLFOX regimen. However, the response to FOLFOX-based chemotherapy remains limited, with complete response rates ranging from 1.8% to 2.4% and partial response rates ranging between 41.1% and 42.5% [6,7]. One of the key factors contributing to the low response rates to FOLFOX therapy is the development of chemoresistance during treatment [8]. Several mechanisms have been implicated in the development of chemoresistance, mainly induction of epithelial–mesenchymal transition (EMT), the production of cancer stem cells (CSCs), and drug efflux mediated by multi-drug resistance (MDR) proteins [9]. In recent years, many molecular markers associated with these processes have been identified. However, considering the extensive number of cellular pathways involved in each mechanism, it is plausible that additional molecular determinants of chemoresistance in GC remain to be characterized.

SOX5 is an 84 kDa transcription factor belonging to the SRY-box transcription factor (SOX) family of proteins, which plays a critical role in cell fate determination during embryonic development and cellular differentiation [10]. Over the past decade, this member of the SOX family has been the focus of numerous studies due to its involvement in oncogenic processes across various cancer types [11]. In breast cancer cell lines, SOX5 has been identified as a promoter of cell proliferation and invasion through the activation of EZH2 [12]. Furthermore, upregulation of SOX5 has been implicated in promoting metastasis in lung cancer [13], hepatocellular carcinoma [14], and prostate cancer [15]. In the context of chemoresistance, SOX5 transactivation has been linked to increased resistance to chemotherapy in lung cancer cell lines [16]. In GC, the role of SOX5 remains underexplored, with recent research suggesting that higher SOX5 expression may be associated with metastasis through the activation of Twist, an EMT regulator and marker [17]. However, studies directly addressing the relationship between SOX5 and chemoresistance to clinical therapies in GC are currently limited.

Although several mechanisms underlying chemoresistance have been described in GC, the upstream transcriptional regulators coordinating these pathways remain poorly understood. This study aimed to evaluate whether SOX5 expression is associated with key biological processes/factors linked to chemotherapy resistance, including EMT, CSC-like phenotypes, MDR, and DNA repair mechanisms. To this end, SOX5 expression and chemoresistance-related markers were analyzed in two GC cell lines representing primary and metastatic disease. Furthermore, cellular sensitivity to 5-fluorouracil and oxaliplatin was assessed through viability assays, and the clinical relevance of SOX5 expression was explored using publicly available data from GC cohorts.

## 2. Results

### 2.1. Detection and Quantification of the Transcription Factor SOX5 in Gastric Cancer Cell Lines

The relative levels of *SOX5* mRNA were quantified by real-time quantitative PCR (RT-qPCR), revealing significantly higher expression in AGS cells compared to MKN-45 cells (Figure 1A). Consistent with these findings, Western blot analysis demonstrated higher SOX5 protein expression in AGS cells relative to the metastasis-derived MKN-45 cell line (Figure 1B). Nuclear localization of SOX5 was confirmed by immunofluorescence analysis in both cell lines (Figure 1C). Taken together, these results demonstrate that AGS cells exhibit higher SOX5 expression at both the transcriptional and protein levels compared to MKN-45 cells.

To extend these findings beyond established cell lines, SOX5 expression was additionally examined by immunohistochemistry in human GC tissue. SOX5 was predominantly detected in the nucleus, with diffuse cytoplasmic staining, in basal epithelial cells within the tumor tissue; some stromal cells were also positive for SOX5 (Supplementary Figure S1). These findings support the clinical relevance of SOX5 expression in GC and are consistent with the nuclear localization observed using immunofluorescence in the AGS and MKN-45 cell lines.

### 2.2. Expression of Epithelial–Mesenchymal Transition Markers in Gastric Cancer Cell Lines

Following the assessment of SOX5 expression, EMT-related markers were analyzed to investigate whether elevated SOX5 levels were associated with a more aggressive GC phenotype. The expression of the EMT transcription factors ZEB1 and Snail1 was evaluated by RT-qPCR and Western blotting (Figure 2A–C). Both markers were significantly increased in AGS cells, which also exhibited higher SOX5 expression at both the transcript and protein levels, suggesting an association between elevated SOX5 expression and activation of EMT-related pathways.

The expression of the epithelial marker E-cadherin, a glycoprotein involved in cell adhesion and strongly associated with a less metastatic and invasive phenotype in various cancers, was also evaluated. A significantly reduced expression of *CDH1* mRNA and E-cadherin protein was observed in AGS cells (Figure 3A,B). A higher accumulation of E-cadherin was observed near the cell surface in the MKN-45 line, while a weaker signal was detected in the AGS line based on immunofluorescence analysis (Figure 3C). Notably, AGS cells, which displayed elevated SOX5, Snail1, and ZEB1 expression, showed a marked reduction in E-cadherin levels, a distinctive feature of cells undergoing EMT, further supporting the association between SOX5 overexpression and a more aggressive cell phenotype.

### 2.3. Expression of Cancer Stem Cell Markers in Gastric Cancer Cell Lines

The levels of two cell surface antigens, CD44 and CD133, which are widely used CSC markers in GC, were assessed [18,19]. Regarding mRNA expression, higher *CD44* expression levels were observed in MKN-45 cells, while *CD133* was expressed at higher levels in the AGS cell line (Figure 4A). Regarding protein expression levels, higher expression of CD133 was identified in AGS cells compared with MKN-45 cells (Figure 4B,C).

### 2.4. Expression of mRNA and Proteins Associated with Chemoresistance in Gastric Cancer Cell Lines

Given the established role of DNA repair mechanisms in chemotherapy resistance [20,21], the expression of two key DNA repair proteins (XRCC1 and ERCC2), together with their corresponding transcripts, was evaluated. Both *XRCC1* and *ERCC2* were significantly upregulated in AGS cells compared with MKN-45 cells at both the mRNA (Figure 5A) and protein levels (Figure 5B,C), which could explain the greater DNA repair capacity in AGS cells, which also exhibited higher SOX5 expression.

In addition, the expression of ABCB1, which encodes the drug efflux transporter P-glycoprotein (P-gp), was assessed by RT-qPCR. AGS cells displayed significantly higher ABCB1 transcript levels than MKN-45 cells (Supplementary Figure S2). As a member of the ATP-binding cassette (ABC) transporter family, P-gp functions as an efflux pump that reduces intracellular drug accumulation and has been widely implicated in the development of chemoresistance across multiple cancer types [22].

### 2.5. Evaluation of Chemoresistance in Gastric Cancer Cell Lines According to SOX5 Levels

Cell viability assays were performed to evaluate the sensitivity of both GC cell lines to 5-fluorouracil (5-FU) and oxaliplatin (Ox), two chemotherapeutic agents commonly used in GC. Following exposure to increasing concentrations of 5-FU for 48 h, AGS cells showed significantly greater viability than MKN-45 cells at most concentrations tested, although this difference was not observed at the highest dose (100 μM) (Figure 6A). Similarly, treatment with increasing concentrations of Ox resulted in greater viability in AGS cells at 20 and 40 μM, while no significant differences were detected at higher concentrations (Figure 6B). To further assess the response to the combined treatment, both cell lines were simultaneously exposed to increasing concentrations of 5-FU and Ox. Under these conditions, AGS cells consistently showed greater viability than MKN-45 cells after 48 h of treatment (Figure 6C). Overall, these findings suggest that AGS cells, which exhibit the highest levels of SOX5 expression, show reduced sensitivity to 5-FU- and Ox-based chemotherapy.

### 2.6. Association of SOX5 Expression with Overall Survival in Gastric Cancer Patients

To investigate the clinical relevance of SOX5 expression, publicly available GC datasets were analyzed using the R2 Genomics Analysis and Visualization Platform. For the survival analysis, overall survival (OS) was analyzed using Kaplan–Meier curves (log-rank method). The approach reported in other publications was used [23,24]. Figure 7A–C illustrate that OS is reduced in groups with high SOX5 expression. In the R2_Nebozhyn and R2_TCGA cohorts, the Bonferroni correction yielded statistically significant results; however, this significance was not observed in the R2_Tan cohort. This discrepancy may be attributed to the smaller sample size and shorter follow-up period in the R2_Tan cohort.

## 3. Discussion

In this study, we investigated the role of the SOX5 transcription factor as a marker of chemoresistance in GC at the transcriptional and protein levels, and its association with clinical survival. To this end, we used the AGS and MKN-45 cell lines, which exhibit high and low expression of SOX5, respectively. Not only was an increase in the expression of SOX5 at the mRNA and protein levels observed, but also stronger preferential nuclear localization. Previous studies have reported that SOX5 overexpression may be associated with a poor prognosis and metastasis in patients [25]. Furthermore, another family member, SOX4, is linked to the development of an aggressive phenotype in GC [26], while SOX5 expression is directly associated with a mesenchymal and aggressive phenotype in GC tissues [13].

The higher SOX5 expression in the AGS cell line—which represents a primary tumor or intestinal-type cell—compared with the MKN-45 cell line, which has greater invasive capacity and an undifferentiated phenotype, may seem contradictory [27]; however, given the plasticity of the tumor process, this finding may be associated with the intermediate phenotypes that have been reported during the epithelial–mesenchymal transition. It has been repeatedly described that cells change their phenotype depending on the stage of disease progression. This process of phenotypic plasticity involves the reprogramming of mesenchymal cells that have migrated from the primary tumor to secondary sites, transforming them into cells with an epithelial phenotype, which facilitates organ colonization and promotes metastatic tumor growth [28,29]. The expression of SOX5 in AGS cells may represent an intermediate step in the generation of cells with a more invasive phenotype, similar to that of MKN-45 cells [30].

To assess the mesenchymal differentiation of the cell lines, the expression of canonical EMT markers was determined. The results show that the AGS cell line, which exhibits higher SOX5 expression, displays significantly elevated levels of ZEB1 and Snail1 at both the transcriptional and protein levels. It is well documented that both proteins (which are transcription factors) are key regulators of cellular reprogramming, enabling the transition from an epithelial to a mesenchymal phenotype, resulting in morphologically elongated, fibroblast-like cells [31]. This transformation increases the mobility and invasive capacity of cancer cells [32], facilitating the spread of cells from the primary tumor to distant sites in the body. Furthermore, when the expression of E-cadherin—which is essential for maintaining polarity and cell adhesion [33]—was examined in the cell lines studied, reduced levels were found to be associated with cells exhibiting a more elongated, fibroblast-like phenotype [31]. In this study, there was higher E-cadherin expression in the MKN-45 cell line, which also exhibited lower levels of mesenchymal proteins, consistent with the findings for canonical EMT markers. Furthermore, the surface antigens associated with the CSC phenotype, CD44 and CD133, are widely recognized as markers of aggressiveness and chemoresistance in various types of cancer [34]. The AGS line showed higher expression of CD133, which is associated with a poor prognosis and chemoresistance in GC. Previous studies on tumor tissues have shown that high CD133 expression correlates with a poorer prognosis in patients with GC [18]. In contrast, the MKN-45 cell line exhibited higher expression of CD44, which is consistent with its long-standing characterization as a CD44+ model for studies on GC therapy [35]. This apparent divergence—lower CD44 in the more chemoresistant AGS line—is not necessarily contradictory since CD44 and CD133 can mark partially non-overlapping stem-like subpopulations in gastric cancer, and their relative contribution to chemo-resistance may differ depending on the cellular context; in AGS cells, CD133 rather than CD44 may be the dominant stemness-associated determinant linked to the high-SOX5 phenotype, whereas MKN-45 cells may rely on CD44-associated mechanisms independent of SOX5 expression. Direct comparison of the functional contribution of each marker to drug resistance in these lines was beyond the scope of the present study.

With regard to MDR phenotype, the AGS cell line showed increased expression of P-glycoprotein (P-gp) transcripts, together with elevated levels of the DNA repair proteins XRCC1 and ERCC2. P-gp has been associated with drug efflux and a poor prognosis in GC due to treatment resistance [36], particularly to platinum-based treatments such as Ox and cisplatin [37]. Elevated expression of ERCC2 has been linked to reduced efficacy of FOLFOX in GC patients with locally invasive or metastatic disease, contributing to chemoresistance [38]. Similarly, the recruitment of XRCC1 to sites of cisplatin-induced DNA damage may inhibit apoptosis, thereby further promoting resistance to treatment [39].

The findings of this study link the presence of SOX5 with increased mesenchymal phenotype and chemoresistance. However, the observed effects on chemoresistance may not be directly attributable to elevated levels of SOX5 expression. The precise mechanisms through which SOX5 might contribute to EMT activation remain to be elucidated. Similarly, how SOX5 induces the expression of CSC markers needs to be identified. Furthermore, one avenue that requires further investigation is how the activation of DNA repair pathways and multidrug resistance occurs. Nevertheless, existing database studies suggest that SOX5 may act as a key mediator of a more aggressive GC phenotype. This hypothesis is supported by in vitro cell viability assays, which demonstrated reduced sensitivity to 5-FU and Ox in AGS cells, the cell line exhibiting the highest expression of SOX5. It is therefore plausible that SOX5 could serve as a marker of chemoresistance, identifying a GC phenotype associated with increased EMT and CSC characteristics. Further studies are required to clarify whether increased chemoresistance is a direct consequence of increased SOX5 expression to fully understand the role of SOX5 in the prognosis of GC and its potential as a therapeutic target (see Study Limitations Section).

Taken together, our findings suggest that high SOX5 expression in GC is associated with a molecular program coordinated by the activation of EMT, the acquisition of characteristics similar to those of CSCs, an increased capacity for DNA repair, and higher expression of genes related to drug efflux. SOX5 expression may be involved in cellular plasticity and adaptation to cytotoxic stress in GC cells following FOLFOX therapy.

### Study Limitations

This study has some limitations that should be considered when interpreting the results. First, the associations described here are based on a comparison of only two GC cell lines, AGS and MKN-45, which differ not only in SOX5 expression but also in their tissue of origin, differentiation state, and genetic background; therefore, some of the phenotypic differences observed between these lines cannot be unequivocally attributed to SOX5. Second, this study did not include gain- or loss-of-function experiments, which are necessary to establish a direct causal role of SOX5 in EMT activation, stemness, DNA repair capacity, or chemoresistance; therefore, the relationships described here should be interpreted as associative, not mechanistic. Third, the clinical relevance of SOX5 was inferred from its association with overall survival in public cohorts and from immunohistochemical detection in gastric tumor tissue (Supplementary Figure S1); a quantitative correlation between SOX5 expression and treatment response in a dedicated patient cohort was not calculated and remains an important direction for future work.

## 4. Materials and Methods

### 4.1. Cell Culture and Reagents

The human gastric adenocarcinoma cell lines AGS (primary tumor; ATCC; CRL-1739^TM^) and MKN-45 (liver metastasis; DSMZ; ACC 409^TM^) were used. Both were cultured in complete RPMI-1640 medium (Gibco, Waltham, MA, USA) with 10% fetal bovine serum (FBS) and maintained in a humidified incubator with 5% CO_2_ at 37 °C. The chemotherapeutic agents 5-fluorouracil and oxaliplatin were purchased from Sigma-Aldrich (Burlington, MA, USA).

### 4.2. RNA Isolation and RT-qPCR

Total RNA was extracted from the GC cell lines using a protocol that uses TRIzol (Ambion Technologies, Austin, TX, USA) and chloroform [40]. The purity of the extracted RNA was determined using a Synergy HTX Multimode Reader Spectrophotometer (Biotek, Shoreline, WA, USA). A total of 2000 ng of cDNA was synthesized using the All-In-One 5X RT MasterMix (ABM, New York, NY, USA) following the manufacturer’s instructions. The cDNA was amplified by RT-qPCR using the Brilliant II SYBR Green RT-qPCR Master Mix kit (Agilent Technologies, Inc., Santa Clara, CA, USA) according to the manufacturer’s protocol. For RT-qPCR, the thermocycling conditions were set as follows: initial denaturation at 95 °C for 10 min, followed by 40 cycles of denaturation at 95 °C for 30 s, annealing at 60 °C for 15 s, and extension at 72 °C for 15 s. The housekeeping gene GAPDH was utilized as a normalizer and the results were analyzed using the 2^−ΔΔCq^ method. The primers for *SOX5* and EMT (*CDH1*, *Snail1*, and *ZEB1*), MDR (*ERCC2* and *XRCC1*), and CSC (*CD44* and *CD133*) markers utilized in the PCR were designed and purchased from Integrated DNA Technologies (IDT-USA, Newark, NJ, USA). The sequences of the primers are provided in Supplementary Table S1.

### 4.3. Western Blotting

Total cellular protein was extracted using RIPA buffer containing a protease inhibitor cocktail (Roche Diagnostics, Indianapolis, IN, USA). Protein quantification was subsequently performed using the Bradford Protein Assay (Sigma-Aldrich, USA). A total of 30 µg of protein was loaded per well in a 10% SDS-PAGE gel and transferred onto a nitrocellulose membrane. The membrane was blocked with a 5% milk solution prepared in TBS-Tween buffer for 90 min at room temperature. The membrane was incubated at 4 °C overnight with a primary antibody diluted in blocking buffer at dilutions of 1:500 or 1:1000 according to the manufacturer’s protocol. The following primary antibodies were used: SOX5 (Abcam, #ab94396, Waltham, MA, USA), Snail1 (Cell Signaling, #3879S, Danvers, MA, USA), ZEB1 (E-Bioscience, #4974182, San Diego, CA, USA), E-cadherin (BD Biosciences, #610181), CD133 (Invitrogen, #PA5-38014), CD44 (Abcam, #ab6124), ERCC2 (XPD) (Cell Signaling, #11963S), XRCC1 (Cell Signaling, #2735S), and β-Actin (Immuno OTM, #691001, Palo Alto, CA, USA). Bound primary antibodies were detected using HRP-conjugated secondary antibodies, which the membrane was incubated with for 1.5 h at room temperature. Detection was carried out using an enhanced chemiluminescence detection kit for HRP (EZ-ECL; Biological Industries, Bilthoven, Kibbutz Beit Haemek, Israel). Chemiluminescence signals were visualized using the Fusion FX imaging system (Vilber Lourmat, Collégien, France), and the optical density of the bands was analyzed using ImageJ software v1.51 (National Institutes of Health).

### 4.4. Immunofluorescence

MKN-45 and AGS cells were seeded onto 12 mm diameter glass coverslips at a confluence of 60–70%. After 48 h, the cells were fixed with a fixing solution (4% paraformaldehyde) for 30 min at room temperature, washed and blocked with 3% BSA (Winkler, Ltd., Stuttgart, Germany) in PBS–glycine for 30 min at room temperature. The cells were incubated overnight at 4 °C with primary antibodies against SOX5 and the various EMT and CSC markers (1:200 dilution). After washing 3 times, they were incubated with Alexa Fluor 633 secondary antibodies for 30 min at 37 °C. DAPI (dilution, 1:10,000; cat. No. sc3598; Santa Cruz Biotechnology, Inc., Dallas, TX, USA) was used for nuclear staining; the cells were incubated with DAPI for 7 min at room temperature. Images of the cells were obtained using a Leica D2500 fluorescence microscope (Leica Microsystems GmbH, Wetzlar, Germany) and a confocal microscope (LSM 510, Zeiss, Jena, Germany).

### 4.5. Cell Viability Assay

Cell viability was assessed using the MTT assay. Briefly, cells at 90% confluence were seeded at a density of 1.5 × 10^3^ cells/well into 96-well plates. After a 24 h incubation in complete RPMI medium supplemented with 10% FBS, the cells were treated with varying concentrations of 5-FU (0, 12.5, 25, 50, and 100 µM) and Ox (0, 20, 40, 60, and 80 µM) for 48 h. In combination experiments involving both chemotherapeutic agents, the specific concentrations of each mixture are detailed in the corresponding figure. Subsequently, 100 µL of MTT reagent (Sigma, Ronkonkoma, NJ, USA), diluted in RPMI medium at a ratio of 1:10, was added to each well. The cells were then incubated for 4 h at 37 °C in the dark. After incubation, the medium was removed, and the resulting formazan crystals were solubilized with 100 µL of a DMSO/isopropanol mixture. The cells were incubated for an additional 15 min at room temperature. Absorbance was measured at 570 nm using a Synergy HTX Multimode Reader Spectrophotometer (Biotek, Winooski, VT, USA).

### 4.6. Bioinformatics Analysis for SOX5 in Clinical Setting

To characterize the clinical role of SOX5, overall survival analysis was performed using the R2 Genomics Analysis and Visualization Platform, a web-based tool validated for use with public databases (http://r2.amc.nl, accessed on 27 March 2026). For the analysis, the following databases were used: “Mixed Gastric-Nebozhyn-400-MAS5.0-u133p2” (GSE66229) [41], “Tumor Stomach adenocarcinoma-TCGA-415-rsem-tcgars”, and “Tumor Gastric-Tan-192-fRMA-u133p2” (GSE15459) [42].

### 4.7. Statistical Analysis

Data analysis was performed using GraphPad Prism 6.0 software (GraphPad Software, Inc). The results are expressed as the mean ± standard deviation (SD) from at least four independent biological replicates (n = 4), each performed on a separate cell passage/culture. Normality of data distribution was assessed prior to selecting the statistical test; given the small sample size per group, the non-parametric Mann–Whitney U-test was used as the primary test for comparisons between AGS and MKN-45 cells, with *p* ≤ 0.05 considered statistically significant.

## 5. Conclusions

Our study establishes a robust framework for future investigations to define the biological and clinical relevance of SOX5 in GC. While the present work identifies a consistent association between SOX5 expression, an aggressive molecular phenotype, and reduced sensitivity to FOLFOX-based chemotherapy, future studies integrating functional genomic approaches, additional experimental models, and clinical validation cohorts are needed to further clarify the role of SOX5 within the complex regulatory networks underlying chemoresistance. Finally, these investigations may contribute to the development of SOX5 as a clinically relevant biomarker for patient stratification and as a promising therapeutic target in GC.

## Figures and Tables

**Figure 1:**
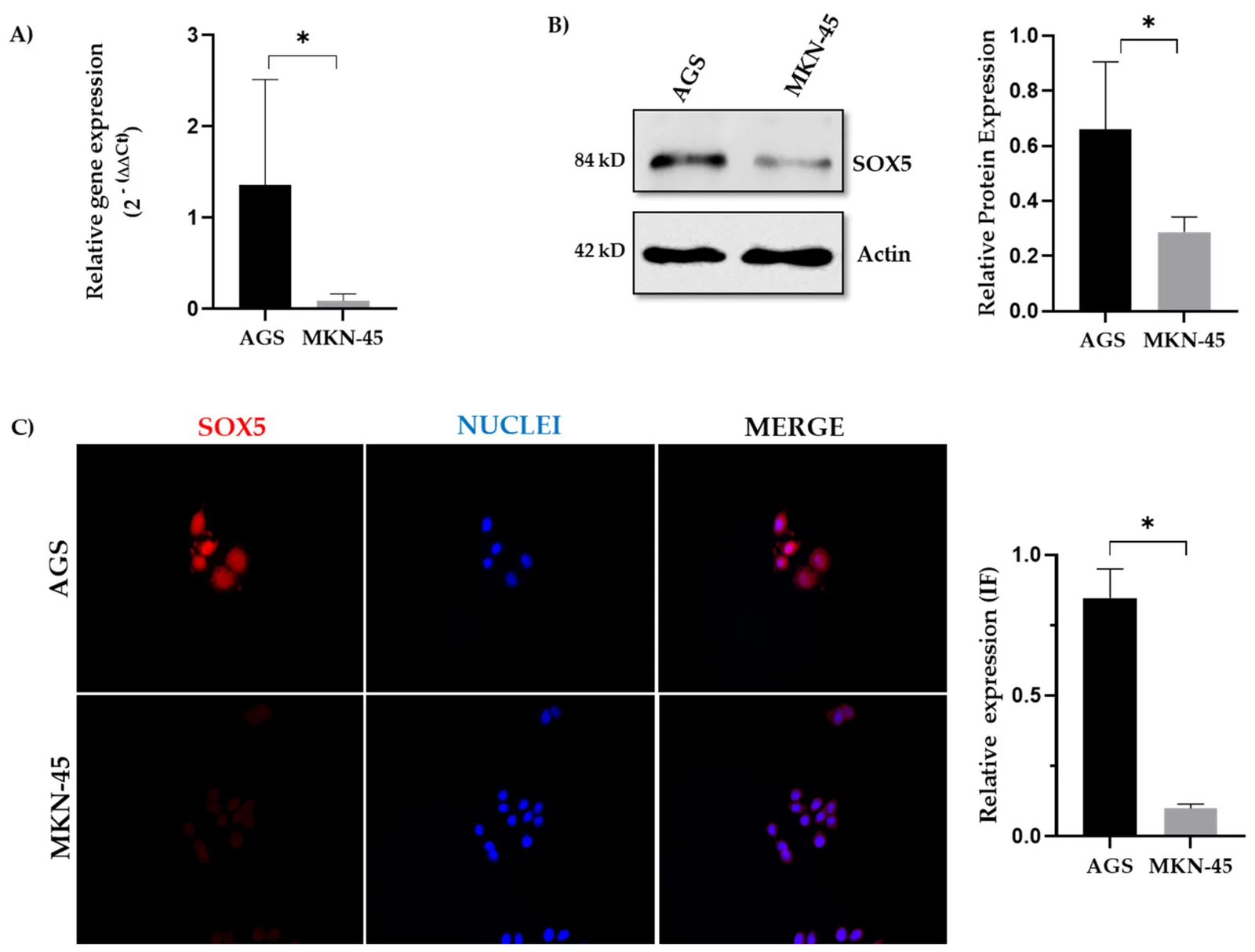
Expression and localization of SOX5 in two gastric cancer cell lines. (**A**) Relative mRNA expression of *SOX5* assessed by RT-q PCR in the gastric cancer cell lines AGS and MKN-45. GAPDH was used as a reference gene. (**B**) Quantification of relative protein expression of SOX5 in AGS and MKN-45 cells. A representative image of the Western blot showing SOX5 and the housekeeping gene β-Actin. (**C**) Representative immunofluorescence images showing detection of SOX5 (red) and DAPI (blue) labeling of nuclei in AGS and MKN-45 cell lines at 40× magnification. Quantification of the co-localization between SOX5 and the nucleus is shown below the images. Data are expressed as the mean ± SD. Mann–Whitney U test was used. * *p* ≤ 0.05.

**Figure 2:**
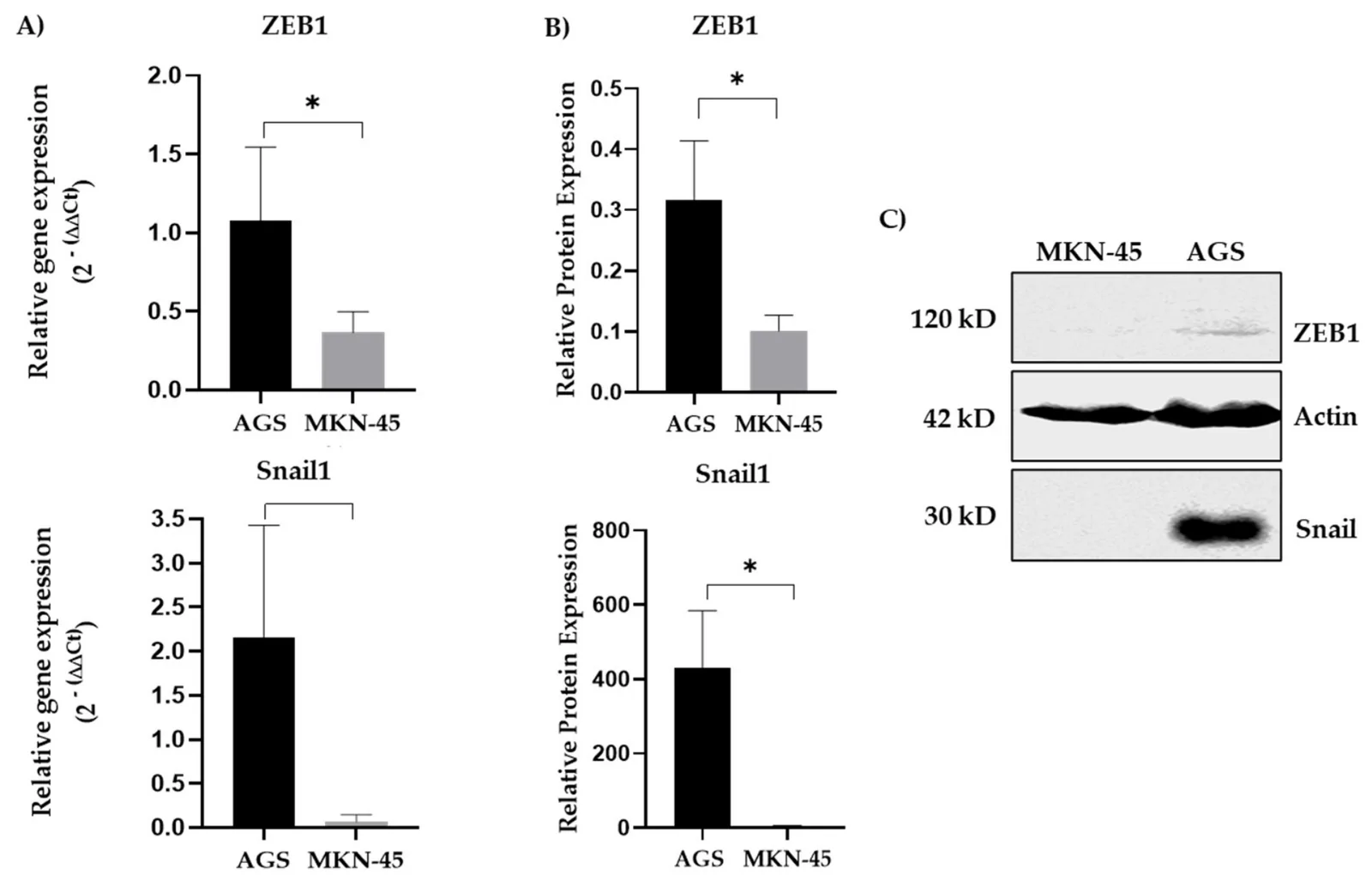
Expression of epithelial–mesenchymal transition markers in two cancer cell lines. (**A**) Relative mRNA expression of Snail1 and ZEB1 measured via RT-qPCR in both cell lines. GAPDH was used as a reference gene. (**B**) Quantification of relative protein expression of Snail1 and ZEB1 in AGS and MKN-45 cell lines. The housekeeping gene β-Actin was used as the reference gene. (**C**) Representative Western blots for ZEB1 and Snail1 protein expression. Data are expressed as the mean ± SD. * *p* ≤ 0.05 (Mann–Whitney U test).

**Figure 3:**
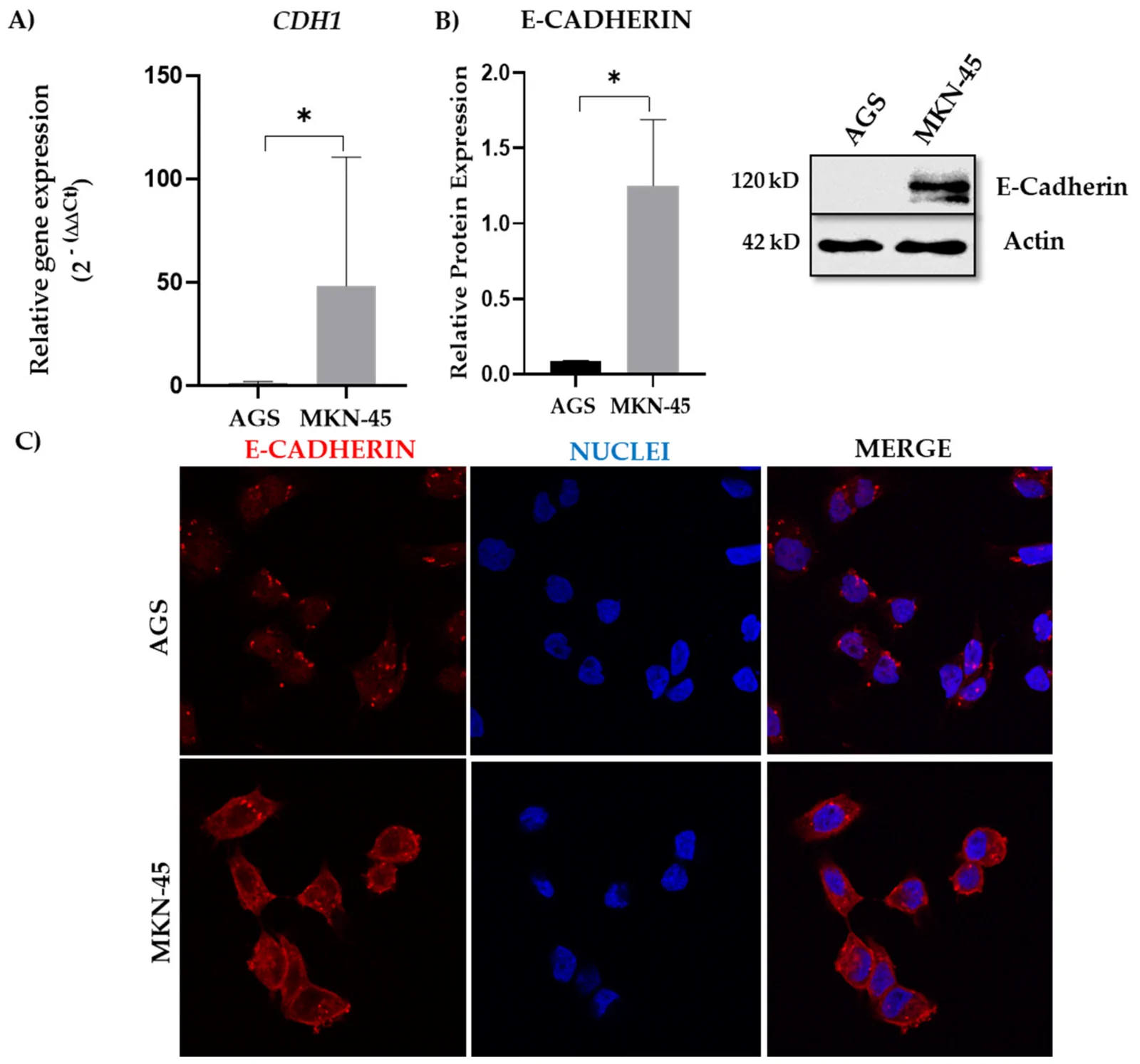
Expression and localization of E-cadherin in two cancer cell lines. (**A**) Relative mRNA expression of E-cadherin (*CDH1*) measured via RT-qPCR in GC cell lines AGS and MKN-45. GAPDH was used as a reference gene. (**B**) Quantification of relative protein expression of E-cadherin in AGS and MKN-45 cell lines. A representative image of the WB for E-cadherin and the housekeeping β-Actin. (**C**) Representative immunofluorescence images showing detection of E-cadherin in both cell lines. Magnification: 40×. Data are expressed as the mean ± SD. * *p* ≤ 0.05 (Mann–Whitney U test).

**Figure 4:**
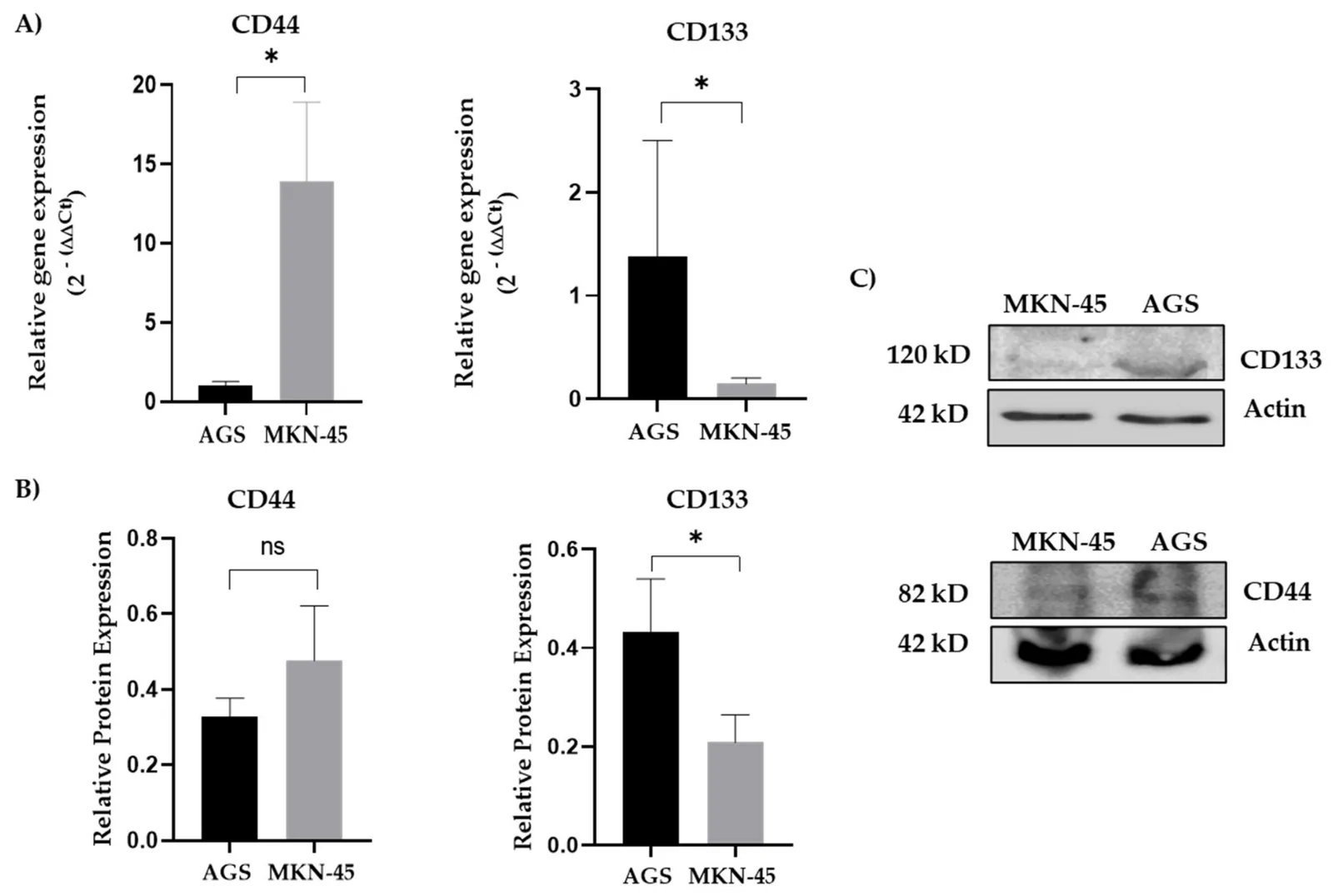
Expression of two cancer stem cell markers in two cancer cell lines. (**A**) Relative mRNA expression of *CD44* and *CD133* measured via RT-qPCR in GC cell lines AGS and MKN-45. GAPDH was used as a reference gene. (**B**) Relative quantification of protein expression of CD44 and CD133 in AGS and MKN-45 cells. The housekeeping gene β-Actin was used as the reference gene. (**C**) Representative Western blots for CD133 and CD44 protein expression. Data are expressed as the mean ± SD. * *p* ≤ 0.05 (Mann–Whitney U test).

**Figure 5:**
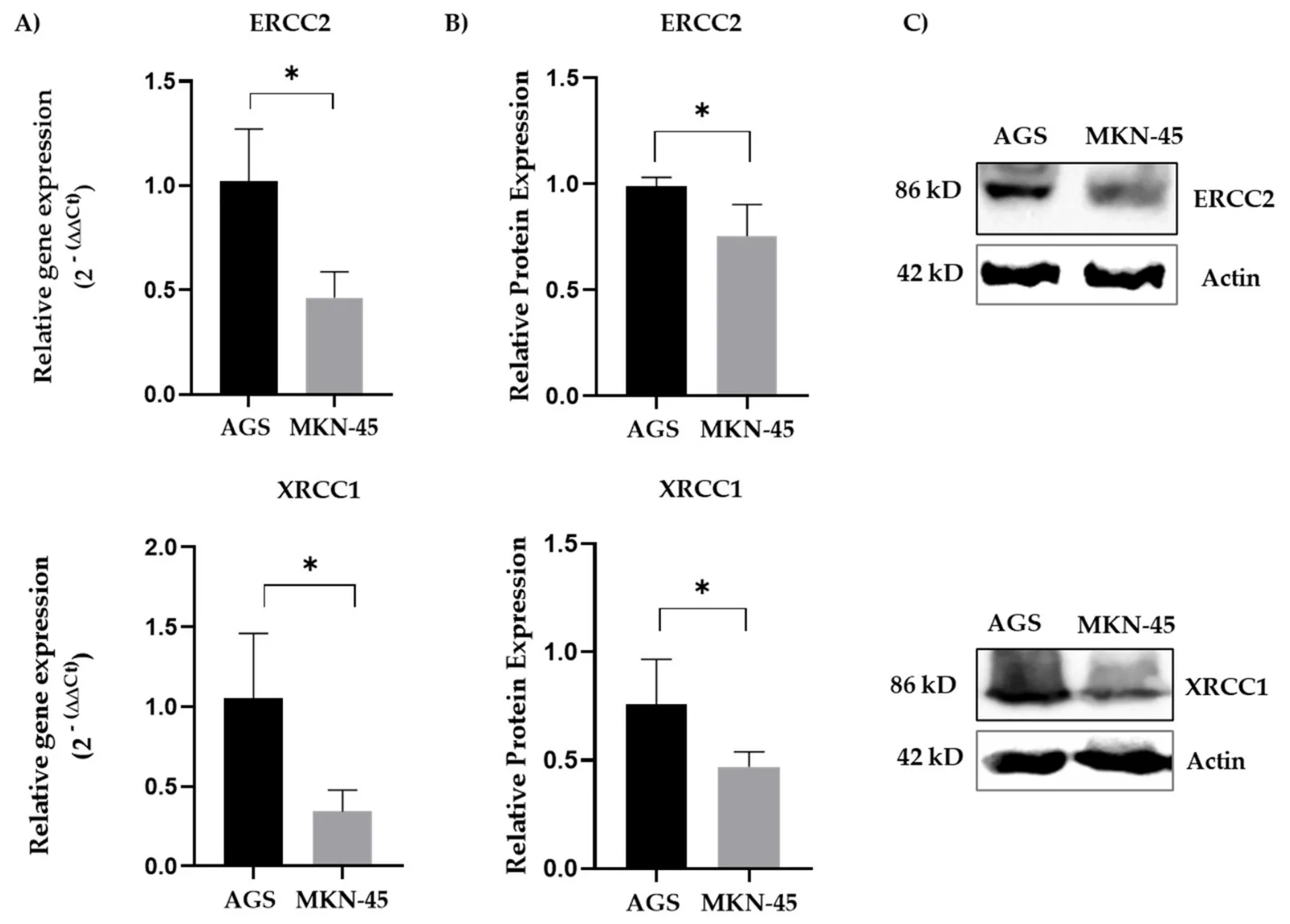
Expression of two DNA repair proteins in two cancer cell lines. (**A**) Relative mRNA expression of *ERCC2* and *XRCC1* measured via RT-qPCR in GC cell lines AGS and MKN-45. GAPDH was used as a reference gene. (**B**) Relative quantification of protein expression of XRCC1 and ERCC2 in AGS and MKN-45 cells. The housekeeping gene β-Actin was used as the reference gene. (**C**) Representative WBs for XRCC1 and ERCC2 protein expression. Data are expressed as the mean ± SD. * *p* ≤ 0.05 (Mann–Whitney U test).

**Figure 6:**
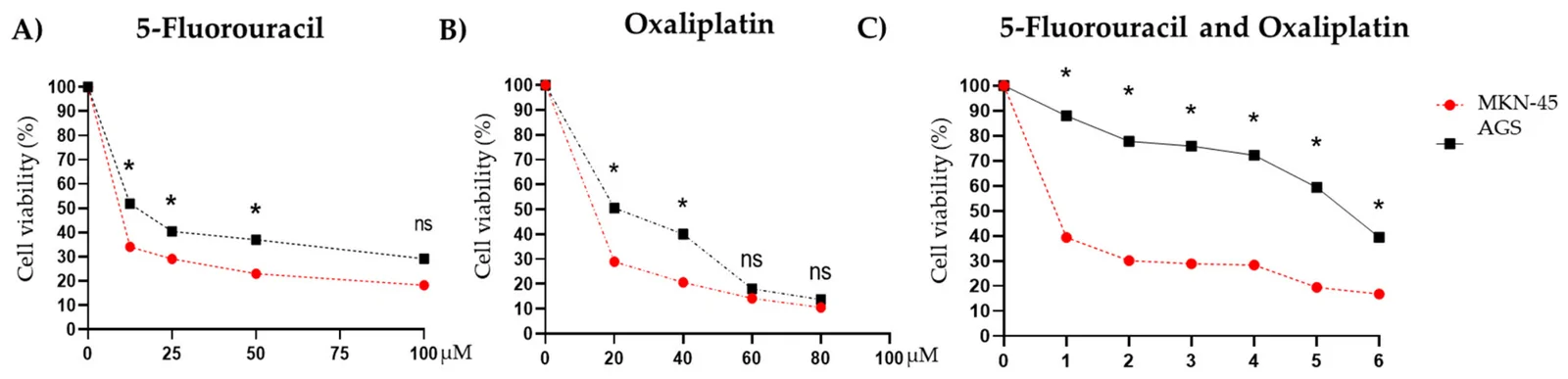
Cell viability assay of two cell lines following treatment with chemotherapeutic agents. (**A**) Cells in exponential growth were treated with various concentrations of 5-fluorouracil (5-FU) (0–100 µM) for 48 h. (**B**) Cells in exponential growth were treated with various concentrations of oxaliplatin (Ox) (0-80 µM) for 48 h. (**C**) Both cell lines were treated with different concentrations of 5-FU (0-100 µM) and Ox (0-60 µM): point 1, 12.5/0; point 2, 12.5/20; point 3, 25/20; point 4, 50/20; point 5, 60/40; and point 6, 100/60. After 48 h of incubation, MTT reagent was used to measure cell viability. Data are expressed as the mean for each concentration combination. * *p* ≤ 0.05 (Mann–Whitney U test). ns: not significant.

**Figure 7:**
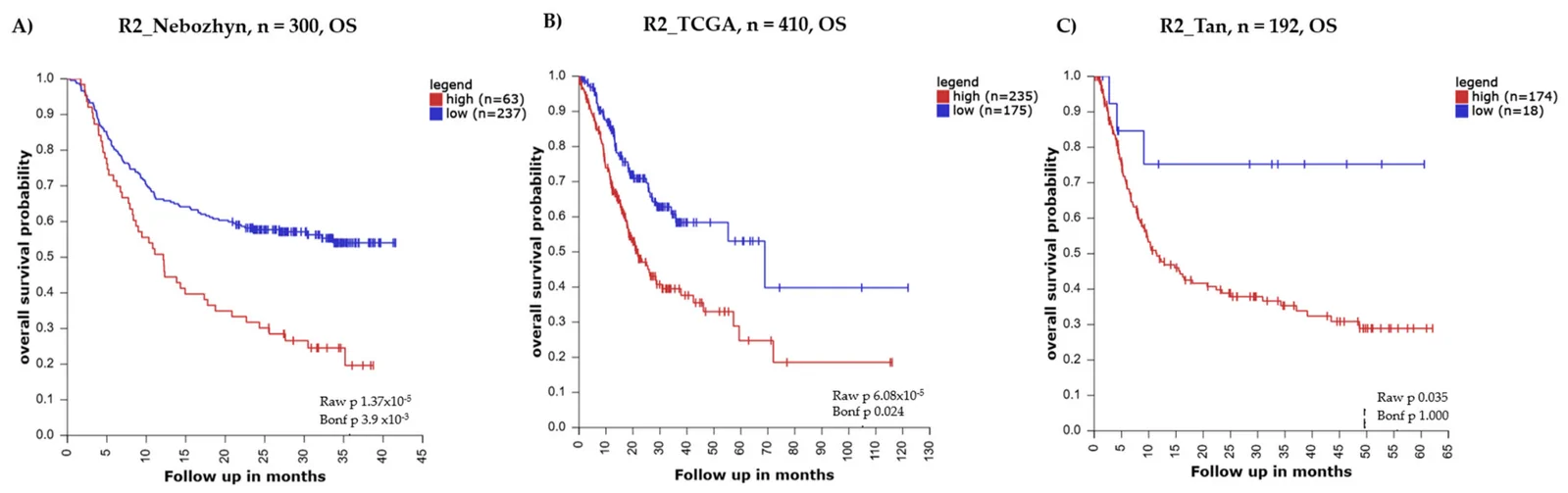
Overall survival in three public gastric cancer datasets using R2 platform (http://r2.amc.nl). (**A**) Overall survival (OS) for 300 patients with gastric cancer (GC) from a compilation of South Korean cohorts. (**B**) OS for 410 patients with GC from The Cancer Genome Atlas (TCGA). (**C**) OS for 192 patients with GC from the Singaporean patient cohort.

## Data Availability

The original contributions presented in this study are included in the article/Supplementary Materials. Further inquiries can be directed to the corresponding authors.

## Supplementary Materials

The following supporting information can be downloaded at: https://www.mdpi.com/article/10.3390/ijms27209228/s1.

## Abbreviations

The following abbreviations are used in this manuscript:

GC	Gastric cancer
FOLFOX	Fluorouracil and oxaliplatin chemotherapy regimen
EMT	Epithelial–mesenchymal transition
CSCs	Cancer stem cells
MDR	Drug efflux mediated by multi-drug resistance
RT-qPCR	Real-time quantitative PCR
ABC	ATP-binding cassette transporter
OS	Overall survival
TCGA	The Cancer Genome Atlas
MET	Mesenchymal–epithelial transition
