# Supplementary material for: The Transcription Factor SOX5 as a Potential Marker of Resistance to Chemotherapy in Gastric Cancer Cell Lines
Source: Int J Mol Sci. 2026 Oct 17;27(20):9228. doi: 10.3390/ijms27209228 (PMC13705015; doi:10.3390/ijms27209228)
Supplement: Supplementary file 1 [file ijms-27-09228-s001.zip › ijms-4466791-supplementary.pdf]

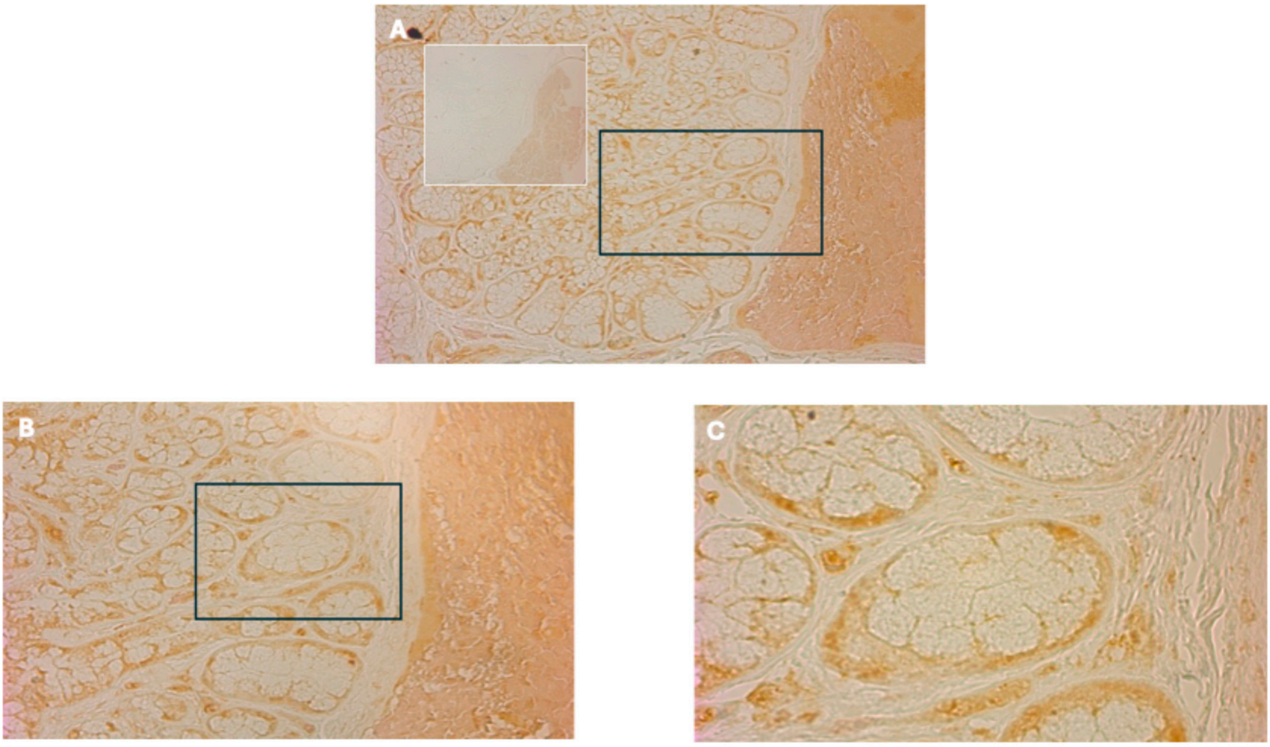


Supplementary Figure S1. SOX5 expression in human cancer gastric. A) Representative image at 200x magnification. The white box is the negative control. The black box indicates the amplified area in B. B) SOX5 expression in the basal zone of the gastric epithelium at 400x magnification. The black box is the amplified area in C. C) Cancer gastric zone amplified at 1000x magnification. SOX5 detection is observed in basal epithelial cells. Distribution is predominantly nuclear and diffuse in the cytoplasm. Some cells present in the stroma are also positive for SOX5.

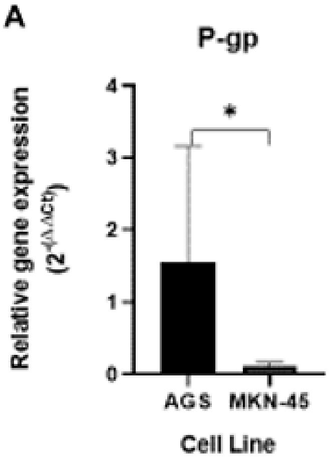


-

Supplementary Figure S2. Transcript expression (mRNA) of ABC transporters in gastric cancer cell lines. (A) Relative mRNA expression of ATP-binding cassette transporters P-gp (transcript for ABCB1), measured via RT- qPCR in gastric cancer cell lines AGS and MKN-45. GAPDH was used as a reference gene. Data are expressed as the mean ± SD (n=4). * $p \leq 0.05$ (Mann-Whitney U test).

**Supplementary Table S1. Primers for RT-qPCR**

| Gene | Forward Primer | Reverse Primer |
|---|---|---|
| ***SOX5*** | 5′-AGGAAGCGGTCGAGACGG-3′ | 5′-CGCCTTCGAAGATGGCGTTG-3′ |
| ***CDH1 (E-cadherin protein)*** | 5′-CGAGAGCTACACGTTCACGG-3′ | 5′-GGGTGTCGAGGGAAAAATAGG-3′ |
| ***ZEB1*** | 5′-GATGATGAATGCGAGTCAGATGC-3′ | 5′-ACAGCAGTGTCTTGTTGTTGT-3′ |
| ***SNAI1*** | 5′-GACGATGACTACGCTTCTGC-3′ | 5′-T TGTATCCTCTTCGGCTGG-3′ |
| ***P-gp*** | 5′-AGCCACGTCAGCTCTGGAT-3′ | 5′-TGCATTCTGGATGGTGGAC-3′ |
| ***CD44*** | 5′-CACGTGGAATACACCTGCCA-3′ | 5′-GACAAGTTTTGGTGGCAGGT-3′ |
| ***CD133*** | 5′-TCAATTTTGGATTCATATTT-3′ | 5′-ACTCCCATAAAGCTGGACCC-3′ |
| ***ERCC2 (XPD protein)*** | 5′-GGCCTCCTGGTATGAGCA-3′ | 5′-CCTGGGTCTCAATAAAGAGCA-3′ |
| ***XRCC1*** | 5′-CTGGGACCGGGTCAAAAT-3′ | 5′-CAAGCCAAAGGGGGAGTC-3′ |
